# Can Desensitizing Toothpastes Also Have an Effect on Gingival Inflammation? A Double-Blind, Three-Treatment Crossover Clinical Trial

**DOI:** 10.3390/ijerph17238927

**Published:** 2020-12-01

**Authors:** Riccardo Monterubbianesi, Scilla Sparabombe, Vincenzo Tosco, Fabia Profili, Marco Mascitti, Andrell Hosein, Angelo Putignano, Giovanna Orsini

**Affiliations:** 1 Department of Clinical Sciences and Stomatology, Polytechnic University of Marche, Via Tronto 10, 60126 Ancona, Italy; r.monterubbianesi@pm.univpm.it (R.M.); s.sparabombe@staff.univpm.it (S.S.); v.tosco@pm.univpm.it (V.T.); fabia.profili@gmail.com (F.P.); m.mascitti@pm.univpm.it (M.M.); a.putignano@staff.univpm.it (A.P.); 2 Department of Clinical and Molecular Sciences, Polytechnic University of Marche, Via Tronto 10, 60126 Ancona, Italy; andrellhosein@hotmail.com

**Keywords:** desensitizing toothpastes, CSPS, CHA, anti-inflammatory, anti-inflammatory activity

## Abstract

Background: Many desensitizing toothpastes exist commercially; however, few clinical trials have investigated their anti-inflammatory effects. This study aimed to evaluate the anti-inflammatory effect and patient appreciation of two toothpastes containing desensitizing agents: (1) a zinc-carbonate-hydroxyapatite nanoparticle (CHA) and (2) a calcium sodium phosphosilicate bioactive glass (CSPS). Methods: CHA and CSPS were compared with an anti-inflammatory and antibacterial herbal based toothpaste (HB). The aims were accomplished by comparing the following outcomes: (1) the reduction in plaque and bleeding score (Full Mouth Plaque Score (FMPS) and Full Mouth Bleeding Score (FMBS), respectively); (2) the antibacterial activity (AbA) of the toothpaste by saliva samples; (3) the patient appreciation score (Visual Analogue Scale; VAS). Clinical parameters were assessed at baseline and 14 days post-treatment. Results: The final sample consisted of 25 subjects, aged between 20 and 58 years. Although no differences in FMPS were reported (*p* > 0.05), both desensitizing toothpastes showed an improvement in FMBS. CSPS and HB recorded more AbA compared to CHA (*p* < 0.05). Moreover, HB resulted in a higher VAS score than both desensitizing toothpastes (*p* < 0.05). Conclusion: In conclusion, only CSPS displayed a similar anti-inflammatory effect compared to HB. Despite the low VAS score, CSPS could be considered as a valid and effective toothpaste in subjects with both dentin hypersensitivity and inflamed gums, highlighting its utility in clinical practice.

## 1. Introduction

Toothbrushing is a complex process, and its efficacy is strongly influenced by the subject’s compliance and manual dexterity. In the developed world, most people would not brush their teeth without using a toothpaste [1], with the primary reason being to have a “good taste” in one’s mouth after daily oral care [2]. However, toothpaste is an ideal vehicle for different agents used to combat several dental diseases: agents to control gingivitis and plaque accumulation, antibacterial agents used to prevent caries and periodontal disease, tooth whitening agents and desensitizing agents used to alleviate dentin hypersensitivity (DH).

DH is a widespread oral health problem among the adult population [3]. It is characterized by an acute passing pain which affects the exposed dentin in response to different stimuli. Dentin, and consequently dentinal tubules, can become exposed after gingival recession or other oral insults, such as attrition or erosion, which ultimately lead to a loss of enamel or cementum [4]. Many theories have been proposed to explain the biological mechanism of DH; however, the hydrodynamic theory is the most widely accepted [3]. According to this theory, pain occurs when a stimulus causes the rapid displacement of fluid within exposed open tubules, which in turn excites nerve terminals at the inner ends of the tubules or the periphery of the pulp [3,5]. Restorative treatment can be performed to alleviate or prevent dentine hypersensitivity. In addition, desensitizing toothpastes can be beneficial in managing dentine hypersensitivity.

The active desensitizing agents found in toothpaste include potassium salt [5], fluorides [6,7], oxalates [7] or bioactive glass [8,9,10,11]. In recent years, desensitizing agents such as calcium sodium phosphosilicate bioactive glass (CSPS) and zinc-substituted carbonate-hydroxyapatite nanostructured microcrystals (CHAs) have been shown to be beneficial even in reducing gingival inflammation [12,13,14,15]. Indeed, toothpastes containing these desensitizing agents, CSPS and CHA, are retailed not only to reduce DH but also gingival inflammation. However, no randomized clinical trial (RCT) has been performed to compare them with an anti-inflammatory toothpaste (herbal based toothpaste (HB)) with proven efficacy. Indeed, the main active natural ingredients of HB reduces plaque accumulation, and consequently, gingival inflammation [16]. For this reason, our double-blind, three-treatment crossover study aimed to compare the anti-inflammatory effect of CSPS and CHA with HB in subjects affected by gingival inflammation.

The aim of the present RCT was accomplished by measuring the differences among three treatment groups at baseline and 14 days post-treatment, in the following outcomes: (1) Full Mouth Plaque Score (FMPS) and Full Mouth Bleeding Score (FMBS) of the participants; (2) the antibacterial activity (AbA) of the toothpastes by evaluating the bacterial vitality in salivary samples; (3) patient’s appreciation using Visual Analogue Scale (VAS). Therefore, the aims of this study were: (1) to evaluate the antiplaque and anti-inflammatory properties and AbA of the tested toothpastes compared to an anti-inflammatory and antibacterial HB; (2) to assess the patient’s appreciation of the tested toothpastes.

## 2. Materials and Methods

This study was performed in the Outpatient Department of Clinical Sciences and Stomatology of the Polytechnic University of Marche, Ancona (Italy) between October 2018 and May 2019. Inclusion criteria were: (1) minimum of 20 teeth; (2) good physical health; (3) aged 18 to 75 years; (4) affected by gingival inflammation for the following conditions: plaque-induced gingivitis in an intact periodontium [17]; plaque-induced gingivitis in a reduced periodontium without a history of periodontitis [17]; plaque-induced gingivitis in a periodontally stable patient [17]; gingival inflammation of periodontitis stage I patients and slow rate of progression [18]; (5) provision of written informed consent. The exclusion criteria were: (1) active caries; (2) periodontitis stage III or IV [18]; (3) wearers of dentures and orthodontic appliances; (4) severe malocclusion; (5) use of oral antiseptics in the previous three months; (6) subjects with medical disorders; (7) undergoing antibiotic or other antimicrobial therapy in previous 6 months; (8) the presence of any systemic disease that could alter the production or composition of the saliva or dental plaque; (9) individuals with an allergy to any ingredients used in the study; (10) smoking; (11) pregnancy.

**Figure 1:**
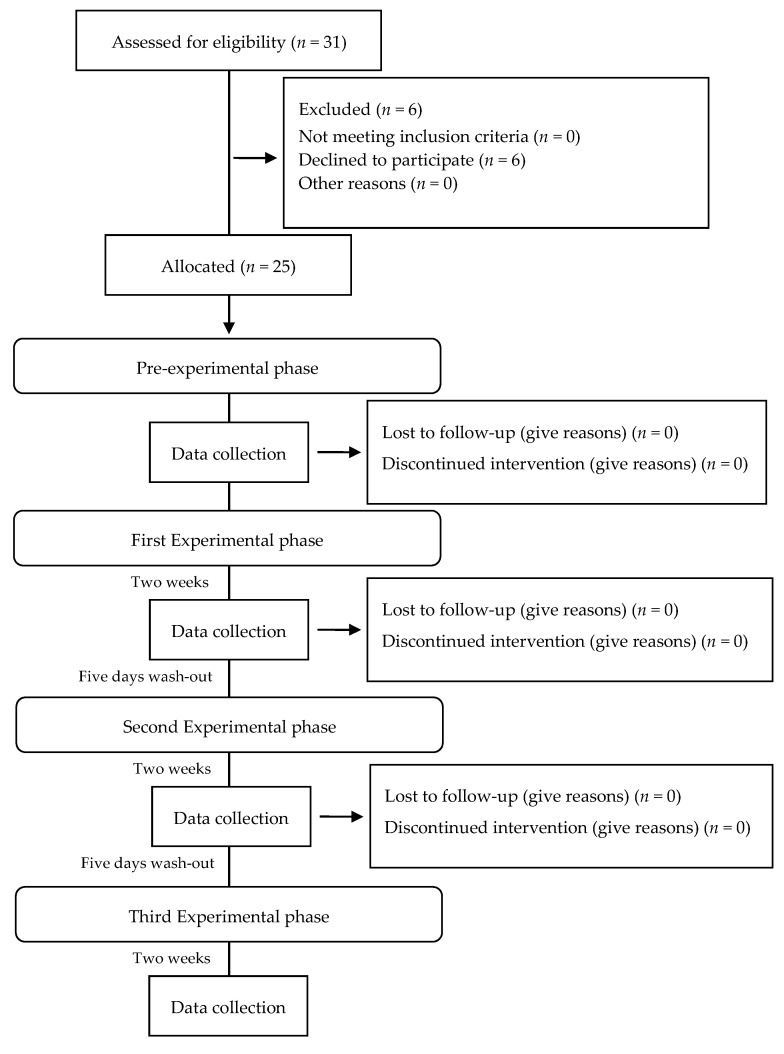
Flow diagram for patient enrollment and study design.

Thirty-one eligible volunteers were recruited for the double-blind three-treatment crossover trial. Six volunteers refused to participate for personal reasons. Twenty-five subjects were used in this study (9 females and 16 males, aged 20 to 58). The protocol consisted of a pre-experimental phase and 3 experimental phases of 14 days, each followed by a 5-day wash-out interval (Figure 1) [19,20,21,22].

In the pre-experimental phase, performed 14 days prior to the experimental phase, subjects underwent a professional oral hygiene procedure, using a blended approach of both mechanical and manual instruments, followed by polishing and flossing in order to eliminate any existing dental plaque and avoid a possible residual effect of other oral hygiene products they may have used. Written instructions and a new toothbrush (Curaprox CS 820 medium, Curaden GmbH, Kriens, Switzerland) were provided to each subject. In the written instructions, the subject was informed to perform toothbrushing 2 times a day [9] with water only, suspending the use of any oral hygiene aids such as mouthwash, dental floss and proxabrush.

After the pre-experimental phase (T0), baseline values of FMPS and FMBS were recorded, and a saliva sample was taken from each subject immediately after all his/her teeth were polished and flossed. The subjects then received the first tested toothpaste and a new toothbrush. Moreover, they were instructed to brush two times a day with a pea-sized dose of the assigned toothpaste. In addition to verbal instructions, the subjects received written instructions. Each experimental phase lasted 14 days and was followed by a 5-day wash-out interval in which the subjects followed the same instruction of the pre-experimental phase. After each experimental phase and before the wash-out interval, FMPS and FMBS were measured and salivary samples were taken, and the patient’s appreciation score was recorded using the VAS. Each experimental phase was assigned a name according to the toothpaste provided. The order of the experimental phases which was randomly chosen was as follows: HB, CSPS, CHA. The tested toothpastes were made indistinguishable by a white label coating that wrapped around the 25 mL tube. Each subject was asked to return all the tubes (used or not used), at the end of each experimental phase. Before each experimental phase, new toothpaste tubes and toothbrushes were provided to the patients, as described in Nogueira-Filho’s study [19]. Additionally, each subject’s teeth were polished and flossed. Oral hygiene instructions and dietary advice was given prohibiting the use of chewing gum and candies in both the pre-experimental and experimental phase.

Two expert dental hygienists (SS and FP) performed the data recording at each oral hygiene session of both pre-experimental and experimental phases. FP was responsible for delivering the anonymous toothpastes while SS was responsible for giving the oral hygiene instructions and recording the data at the end of the pre-experimental and each experimental phase. The order in which toothpastes were provided in this double-blind three-treatment crossover study was determined through a computer-generated random sequence. The investigators were neither involved in the randomization process, nor were they aware of the assigned group in all outcome evaluations. The study was conducted in accordance with the Declaration of Helsinki. Moreover, the protocol was carried out in accordance with the recommendations by the local Ethics Committee (N. 0405OR; 25 February 2016).

### 2.1. Toothpastes

The following two toothpastes containing desensitizing agents were compared with a well-known anti-inflammatory toothpaste:

- CSPS contains 5% calcium sodium phosphosilicate bioactive glass (Sensodyne Protezione Completa, GlaxoSmithKline Consumer Healthcare, Brentford, United Kingdom).
- CHA is a zinc-carbonate-hydroxyapatite nanoparticles toothpaste (Biorepair plus Parodontgel, Coswell S.p.A, Bologna, Italy).
- HB is an herbal anti-inflammatory toothpaste containing sodium bicarbonate (67%), Peppermint, Echinacea, Sage, Myrrh, Rhatany, Chamomile (3%) and sodium fluoride (1400 ppm) (Parodontax, GlaxoSmithKline Consumer Healthcare, Brentford, United Kingdom). It has shown anti-inflammatory activity and therefore was considered as the control [23,24,25,26].

### 2.2. Clinical Assessment

The plaque score and bleeding score of the subjects were measured using the periodontal indices, FMPS and FMBS, respectively. Their clinical assessments were performed on 6 surfaces, as described in the literature [27,28].

The AbA of the tested toothpastes was analyzed by measuring the vitality of salivary bacteria. This was accomplished through 10^−3^ saliva dilution, homogenization using a vortex mixer for 30 s, followed by incubation at 37° for 24 h in Columbia agar +5% sheep blood [29]. The saliva sample was always taken in the morning, before any other assessment, without the subject brushing their teeth or eating. Samples of 2 mL of unstimulated saliva were collected using the spitting method [29]. After each experimental phase, the patient appreciation score (VAS) was assessed by choosing a number on a 10 cm line, with a score of 0 indicating a bad taste and a score of 10 indicating a good taste [30].

### 2.3. Sample Size Examination

Calculation of the number of subjects was centered on the principal outcome i.e., the average decline in the index of plaque between the beginning and the end of treatment. It is estimated that an initial average value of plaque index was equal to 55%. Assuming a relative decrease of 15% of plaque index (i.e., 8% absolute), a standard deviation not dissimilar from baseline (10%), and an intra-cluster average correlation (0.2 considering the wash-out) with an alpha equal to 0.05, the minimum sample size to obtain a power of 80% was 22 subjects [22]. In order to prevent disclaimers, the minimum sample size was estimated at 31 subjects.

### 2.4. Statistical Methods

Statistical analysis was performed with R Project for Statistical Computing 3.3.0 (https://www.r-project.org/) (R Development Core Team, Ames, IA, USA) and Microsoft Excel 2013 (Microsoft, Washington, DC, USA). Statistical evaluations were made only on the subjects who concluded the study. For each clinical outcome, the comparison between the baseline and post-intervention within the three treatment groups was analyzed using the Wilkoxon test, also called “matched-pairs signed ranks test”. The comparison between differences in the pre-post intervention mean, for all continuous outcomes, was carried out by a non-parametric Kruskal–Wallis test. A *p*-value < 0.05 was considered significant for all results.

## 3. Results

Of the 31 recruited subjects, a total of 25 subjects, 9 female and 16 male, age range 20–58 years, completed the trial. Table 1 shows the results of the study.

All the tested toothpastes displayed a similar FMPS (*p* > 0.05). On the other hand, they showed an improvement of FMBS (HB = 0.10 ± 0.04; SPS = 0.09 ± 0.04; CHA = 0.08 ± 0.03) compared to T0 (0.17 ± 0.06) (*p* < 0.05). However, there were no statistical differences between the three toothpastes.

In contrast, different results were observed with regard to AbA. CSPS and HB (6.25 ± 13.48; 4.45 ± 3.06, respectively) were statistically different from T0 (20.49 ± 17.53) (*p* < 0.05). However, CHA (14.41 ± 19.66) was not different from T0, CSPS and HB (*p* > 0.05). Regarding patient appreciation, HB showed a higher VAS score (6.30 ± 2.08) than both CSPS and CHA (3.00 ± 2.08 and 3.05 ± 1.88, respectively). No adverse reactions were observed or reported during the trial. There was no report of discomfort related to the use of tested toothpastes.

## 4. Discussion

Both tested toothpastes, which contain desensitizing agents, CSPS and CHA, are commercially marketed to reduce not only DH but also gingival inflammation. Despite this, there has been no RCT to evaluate the anti-inflammatory activity of CSPS and CHA. Our double-blind three-treatment crossover trial, therefore, aimed to compare the anti-inflammatory activity of the two test toothpastes with HB, as the active control [23,24,25,26,31]. HB has been shown to exert antiplaque and anti-inflammatory activity. It contains naturally occurring substances, mainly sodium bicarbonate, which causes cell wall degradation and an enzymatic inhibition which in turn prevents bacteria from aggregating with Gram-positive pioneer species and reduces bacterial multiplication [16,32]. The effectiveness of HB in reducing gingival inflammation and plaque accumulation has been well reported [33,34]. On the other hand, CSPS and CHA are toothpastes based on desensitizing agents. CSPS contains bioactive glass which, when exposed to body fluids, reacts and deposits hydroxycarbonate apatite on the exposed dentine; the deposited hydroxyapatite has a chemical structure similar to enamel and dentin [14,35,36]. CHA contains microcrystals with a low dissolution rate and releases ions such as Ca ions, phosphates and Zn ions, thus forming a biomimetic coating on the surface, which may remineralize the altered enamel surfaces and close dentinal tubules [15,37].

Since all three groups presented an FMPS value similar to that of the baseline, the tested toothpastes showed no evident antiplaque activity. One explanation of this result could be the so-called “sliding effect” [38]. It was hypothesized that the presence of toothpaste in sufficient amounts on the occlusal surface or the approximal sites could result in a reduction in the shear force of the toothbrush filaments in removing the adhered plaque [39]. Therefore, the present study supports the theory that the mechanical action provided by the toothbrush appears to be the main factor in the plaque removal process [19,38,39,40,41].

Although both CSPS and CHA showed the same reduction in FMBS compared to HB, only CSPS and HB (6.25 ± 13.48; 4.45 ± 3.06, respectively) showed a significant reduction in AbA compared to baseline (20.49 ± 17.53). Therefore, CSPS has been proven to be beneficial in reducing gingivitis. The present findings are only in partial agreement with the study of Aruna et al., which reported that use of a toothpaste containing calcium sodium phosphosilicate led to gingival bleeding reduction compared to a placebo toothpaste; however, a supra-gingival plaque reduction was also reported [12]. Moreover, the present results agree with Tai et al., which also demonstrated that a toothpaste containing calcium sodium phosphosilicate decreased gingival inflammation [42].

The possible AbA mechanism of action of CSPS could be explained by the antibacterial effect of high rates of ions released during the reaction in the oral cavity and the associated local changes in pH [43,44]. On the other hand, although there was a reduction in FMBS, CHA showed no different AbA from baseline. The aforementioned biomimetic coating formed by CHA on the tooth surface may be protective toward bacterial attacks [15,45]. Our finding disagrees with Hanning et al., who demonstrated antibacterial and antiadherent properties of CHA, although a mouth rinse with the same CHA composition was tested instead of a toothpaste [13].

In the present study, VAS was used to evaluate the subject’s appreciation of the different toothpastes. HB was the most appreciated toothpaste (6.30 ± 2.08), while CSPS and CHA (3.00 ± 2.08 and 3.05 ± 1.88, respectively) showed similar yet lower VAS values. Toothpaste taste contributes to a fresh mouth experience which makes brushing an acceptable or even pleasant experience. In several studies, it was observed that brushing without the use of toothpaste was judged as unpleasant and discouraged patients from cleaning their teeth [38,46,47]. Although no article has compared the patient appreciation of the taste of the tested toothpastes, there is a study which has agreed with our findings of high patient appreciation of HB’s taste [48]. Nevertheless, the results of this study should be interpreted with caution, as the sample size is small, and the sampling frame may not represent the general population.

## 5. Conclusions

In addition to their recognized desensitizing activity, both CSPS and CHA showed an anti-inflammatory effect comparable to HB. Moreover, CSPS showed an AbA higher than CHA and comparable to HB. Toothpastes containing CSPS, therefore, to a greater extent than CHA, may represent a clinically effective option for patients who suffer from both DH and gingival inflammation and therefore should be recommended by clinicians. Using one toothpaste instead of two different toothpastes is also a more practical option. Significantly, a toothpaste with a pleasant taste can improve oral hygiene compliance in the young and old. Therefore, an improvement of CSPS taste could be useful in making it more appreciated by patients and consequently, more frequently used. Further studies are warranted to evaluate the efficacy and the antibiotic mechanism of action of 5% calcium sodium phosphosilicate bioactive glass toothpaste.

## Figures and Tables

**Table 1:** Comparison between groups. All values are expressed as means and standard deviations.

	T0	HB	CSPS	CHA
FMPS	0.54 ± 0.14 ^a^	0.55 ± 0.10 ^a^	0.52 ± 0.10 ^a^	0.55 ± 0.09 ^a^
FMBS	0.17 ± 0.06 ^a^	0.10 ± 0.04 ^b^	0.09 ± 0.04 ^b^	0.08 ± 0.03 ^b^
AbA (×10^8^)	20.49 ± 17.53 ^a^	4.45 ± 3.06 ^b^	6.25 ± 13.48 ^b^	14.41 ± 19.66 ^a,b^
VAS		6.30 ± 2.08 ^a^	3.00 ± 2.08 ^b^	3.05 ± 1.88 ^b^

Note: Full mouth plaque score (FMPS), full mouth bleeding score (FMBS), antibacterial activity (AbA) and patient’s appreciation (Visual Analogue Scale; VAS) at baseline (T0) and at the end of each experimental phase. Each experimental phase was assigned a name according to the toothpaste provided, and the order of the experimental phases was as follows: herbal based toothpaste (HB), calcium sodium phosphosilicate bioactive glass (CSPS), zinc-carbonate-hydroxyapatite nanoparticles (CHA). Different superscript letters (^a^/^b^/^a,b^) indicate significant differences between groups (*p* < 0.05).
